# The Effect of Locomotion on the Mobilization of Minerals from the Maternal Skeleton

**DOI:** 10.1371/journal.pone.0122702

**Published:** 2015-03-23

**Authors:** Wendy R. Hood, Michael Hobensack

**Affiliations:** Auburn University, Auburn, Alabama, United States of America; Inserm U606 and University Paris Diderot, FRANCE

## Abstract

Bone is a dynamic tissue from which minerals are deposited or withdrawn according to the body’s demand. During late pregnancy and lactation, female mammals mobilize mineral from bone to support the ossification of offspring skeleton(s). Conversely, in response to mechanical loading, minerals are deposited in bone enabling it to develop a stronger architecture. Despite their central importance to reproductive performance and skeletal integrity, the interactions between these potentially opposing forces remains poorly understood. It is possible that inter-individual differences in the loading imposed by different forms of locomotion may alter the amount of mineral mobilized during reproduction. Here, the impact of vertical versus horizontal locomotion on bone mobilization was examined during reproduction in the laboratory mouse. The vertical, or climbing, group had access to a 60-cm tower, increasing strain on their appendicular skeleton. The horizontal, or tunnel, group had access to a 100-cm tunnel, which encouraged movements within the horizontal plane. Form of locomotion did not impact the amount of bone females mobilized during reproduction or the amount of mineral females deposited in the litter, but maternal bone architecture differed between groups. The climbing group displayed more trabeculae than the tunnel group, whereas the tunnel group displayed greater cortical bone mineral density mid-shaft. Interestingly, pups born to mothers in the climbing group had a higher concentration of total body calcium at 16 days than pups of mothers in the tunnel group. As maternal total body calcium composition and the amount of calcium invested in the full litter were not different between groups, the difference in the relative calcium content of pups between groups is not suspected to reflect difference in mineral allocation. Future research should consider the impact of maternal activity on the efficiency of offspring skeletal ossification via hormones and other bioactive factors transferred in utero and in milk.

## Introduction

Developing vertebrate young require a large quantity of calcium and phosphorus to support the growth and ossification of their skeletons. Until weaning, most young mammals are entirely dependent on their mother for mineral resources. To support this demand, reproductive females in many species of mammals supplement minerals that are ingested with minerals that are mobilized from their own skeleton [1–5]. Loss of mineral affects bone architecture primarily by reducing the number and diameter of cancellous bone trabeculae. The struts formed by trabeculae are a primary determinant of bone strength so loss of trabeculae leaves bones less able to handle loading and strain during locomotion [6]. Cortical thickness can also be affected but usually to a lesser degree [7].

In growing and full-sized adults, bone also displays a plastic response to the loads and stresses that it experiences. The mineral content and structural architecture of bone is enhanced with a long-term increase in load and reduced when loads are lessened [8]. These adjustments allow the individual to adapt to changes in the relative forces that its skeleton must withstand [9,10]. The response of bone to changes in mechanical strain has been characterized in rodents. These studies show that rodents given the opportunity to run display greater cancellous bone formation and cancellous bone volume relative to those maintained in a standard rodent box [11], while rodents given the opportunity to climb have greater bone mineral density, greater trabecular thickness, and greater cortical cross-sectional area in weight-bearing appendicular bones [12,13].

In many eutherian mammals, pregnant and lactating females experience an increase in loading on their skeleton associated with an increase in body mass; these stresses may be particularly high during foraging movements such as running, climbing, digging, swimming and/or flying. A better understanding of the interaction between the anabolic effects of load bearing during locomotion and the catabolic effects of reproduction on bone could lead to better understanding of the regulation of mineral allocation to offspring. If skeletal strength is prioritized over mineral allocation to young, the total amount of mineral that can be transferred to the litter may be reduced. Alternatively, if mineral allocation to young is maintained at the expense of bone strength, a mother’s probability of fracture will increase.

Although these interactions have not been addressed in wild vertebrates, the relationship between exercise and loss of bone mineral has been examined in pregnant and lactating women [14–17]. The results of these studies have produced mixed results. Drinkwater and Chesnut [15] and Little and Clapp [16] reported a lack of a clear response of bone relative to a woman’s activity level. Both sets of researchers attributed the lack of effect to difficulty in controlling women’s diets, exercise regimens, and other daily activities that contribute to mechanical loading on the skeleton. In contrast, under more controlled exercise regimes, Lovelady et al [17] found that aerobic and resistance exercise increased bone mineral density of lumbar vertebrae during lactation. Dimov et al [14] found that playing tennis during gestation increases cancellous bone mineral density. The interaction between loading on the skeleton during locomotion and bone mobilization in reproductive females has not been investigated for other species.

Small mammals transfer high amounts of nutrients to their young and display relatively high levels of bone mobilization during reproduction [18,19]. In addition, the skeleton makes up a smaller proportion of total body mass in small species than large [20], and the relative loss of bone mineral is typically greater in small than large species [19]. Thus, when mineral used for current young comes at a cost to the female’s skeletal integrity, as may be most common in small species (but see [21]), reproductive females that experience high mechanical loading are expected to reduce bone mobilization. This strategy would improve a mother’s fitness by increasing her probability of surviving the current reproductive bout without critical injury and thus, increasing her probability of reproducing again.

Mice and other rodents may walk, run, or climb to acquire sufficient nutrients to support the high demand of reproduction. The relative amount of strain that the skeleton experiences during these movements may impact how much bone can be mobilized to support reproduction. In exercise physiology studies, voluntary climbing in rodents is considered a form of resistance training associated with the loading imposed by the animal’s mass as it climbs [22]. In rats, individuals subject to resistance training display greater bone mineral content and greater trabecular bone formation, and as a result, greater trabecular thickness than sedentary individuals [22] and individuals subject to aerobic training [23]. Whereas, individuals subject to resistance training and individuals subject to aerobic training both display greater cortical bone formation than sedentary individuals. [23]. Thus, we predict that climbing mice will have more numerous and wider trabeculae, greater bone mineral content, and transfer fewer minerals to their young than mice whose movements are limited to the horizontal plane. Sedentary mice are predicted to display reduced cortical thickness relative to climbing mice and mice that walk or run in a horizontal plane, but still display similar bone mineralization and mineral allocation to young to mice that move in a horizontal plane.

Recent work suggests that energy and bone metabolism interact [24–26]. Thus, to examine the effect of impact locomotion on bone mobilization during reproduction, we compared parameters of bone mineralization and mineral deposition in young between mice in cages that required different predicted degrees of skeletal loading and differences in predicted energy expenditure associated with acquiring food. Specifically, we compared mice subjected to A) high loading and high energy expenditure—mice were maintained in boxes with a 60-cm tower that encouraged voluntary climbing, B) low loading and high energy expenditure—mice were maintained in boxes with a 100-cm tunnel that encouraged walking and running in a horizontal plane, and C) low loading and low energy expenditure—mice were maintained in standard mouse boxes where the mice were predicted to be relatively sedentary.

## Materials and Methods

All procedures followed recommendations in the Guide for the Care and Use of Laboratory Animals of the National Institutes of Health. The protocols described herein were approved by the Auburn University Institutional Animal Care and Use Committee (protocol number: 2008-1471).

Virgin outbred mice (*Mus musculus*, ICR-strain) were used for this experiment. The adult females used in this experiment were reared in the Lab Animal Health Facility at Auburn University (all young born to mothers described in [3]). In effort to control for the impact of natal litter size on adult phenotype, all females used in this study were reared in a litter of 8 or 13 young and females from each of these litter sizes were divided equally among treatment groups. Twelve females were assigned to each of 3 treatment groups based on housing design, including tower, tunnel, and box groups as described below. Female mice were initially acclimated to the new box design in groups of 6 mice for 3 weeks, allowing bold mice to train those that were less bold. After three weeks, pairs of females were moved to smaller boxes. Males were added to these boxes for mating 11–28 days later and removed after both females displayed a vaginal plug or when at least one female in the group displayed a significant increase in body mass indicative of pending parturition. Females were moved into individual boxes approximately 1 week before parturition. Females were approximately 12 weeks old at the time of mating. All males were approximately 6 months old and mated previously [3]. Care was taken to ensure that no females mated with their fathers. Animals were maintained on a 14:10 h light cycle at 24±1°C. Food and water was offered *ad lib* (Rodent breeder diet 8626; Teklad Diets, Madison WI; USA, 3.5 kcal/g metabolizable energy, 21.3% protein, 10.5% fat, 3.2% crude fiber, 1.1% calcium, 0.98% phosphorus).

### Box Design

**Fig 1:**
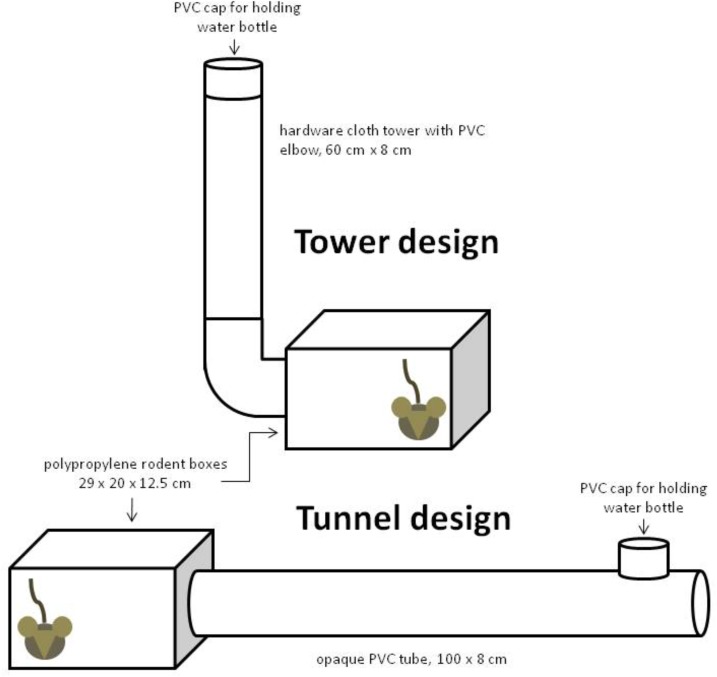
Design of tower and tunnel box used in this experiment. Dimensions and materials are given.

All animals were initially maintained in modified 42 x 21 x 20 cm polypropylene rat boxes for the first 3 weeks and then moved to 29 x 19 x 12.5 cm polypropylene mouse boxes for the rest of the experiment (Lab Products, Inc., Seaford, DE, USA). In all cases, food was placed inside of the box instead of offering it in a wire hopper. Wire hoppers were removed to prevent the mice in the tunnel and box groups from hanging from the hopper, a common behavior that would have placed considerable tension on their skeletons [27]. Animals in the tower group were assigned mouse boxes fitted with a cylindrical hardware cloth tower (0.5 cm^2^ galvanized steel mesh) intended to impose loading on the skeleton (Fig. 1). This design allowed for voluntary climbing that was encouraged by placement of the water bottle at the top of the tower. Animals in the tunnel group were assigned mouse boxes fitted with a PVC tunnel, in an attempt to stimulate walking and running with energy expenditures equivalent to the tower group while maintaining low skeletal loading (Fig. 1). Placement of the water bottle encouraged movement along the length of the tunnel. A third group of mice were assigned standard mouse boxes. With the hopper removed to prevent climbing, an inverted PVC cap was fitted inside of the box to hold the water bottle. All boxes were covered with microisolator lids. The tower and tunnel lids were approximately 5 cm high, whereas the box group lids were 10 cm high, allowing sufficient room for the water bottle to be placed in the box. This design failed to prevent climbing and thus mice could not be considered sedentary. Mice climbed on the water bottle, and hung from and chewed on the box lids. Because this design failed to achieve low skeletal loading, this group was removed from all comparisons.

### Relative Energy Expenditure and Body Composition

As an indicator of relative energy expenditure, the mass of food consumed was determined bi-weekly by weighing the amount of food offered and the amount of residual food 3–4 days later; cumulative intake of individuals was based on the sum of these values during lactation. The body mass of mothers was recorded bi-weekly to determine when they neared late pregnancy and at the termination of the study. All breeding females and their young were euthanized with carbon dioxide at peak lactation (day 16 post-partum). Each mouse was weighed and then stored at −20°C for later analysis. Animals that didn’t reproduce were euthanized within the same time frame to ensure that all animals were similar in age at the termination of the study. All animals had remained in the respective box type for an average of 10 weeks at the termination of the study (range 9–12 weeks).

All female carcasses and 3 randomly selected pups carcasses per litter where thawed and both femurs excised. The remaining carcass for each animal was dried to a constant mass in a forced convection oven (Binder drying oven FED 115-UL, Binder Inc., Great River, NY, USA) at 60°C. Dried tissues were homogenized and subsampled for measurements of total body fat (∼0.5 g for adults and ∼0.3 g for pups) and ash content (∼0.5 g for adults and ∼0.3 g for pups). Two replicates were run for each assay. Neutral lipids were extracted with petroleum ether in a soxhlet apparatus until samples reached constant mass (12 h). The total fat content of each replicate was determined based on the difference in mass between the pre- and post-extracted samples.

The right femur of each mouse was manually cleaned of residual muscle tissue after soaking the bone in an ultrasonic bath for approximately 30 minutes to loosen the attached tissue. Fat was extracted from cleaned bones in the soxhlet, as described previously (see [3] for comments on methodology). The adult femora were photographed against a 1mm^2^ grid under a dissecting microscope and the length of a single square in grid was measured for calibration and the length of each bone was measured using Image J software (Image J, National Institutes of Health, Bethesda, MD, http://imagej.nih.gov/ij/). Pup femora were measured by hand with calipers (0.01 mm). Any residual tissue remaining on the bone following ether extraction was removed prior to ashing.

Whole body homogenates and fat-extracted femora were ashed in a muffle furnace (Fisher Scientific Isotemp Muffle Furnace, Dubuque, IA, USA) at 550°C, with the whole body samples ashed for 12 h and the bones ashed for 24 h. The ash content of samples was determined based on the change in mass following ashing. Ashed samples were digested in 70% trace metal grade nitric acid. The adult femur samples were digested in a microwave digestion unit (Speedwave MWS-2, Berghof Products, Eningen, Germany). The microwave was ramped to 200°C over 15 min, held at 200°C for an additional 15 min, and then ramped back down to room temperature over the final 15 min of each run. Because of an electrical malfunction with the microwave digester, the carcass samples were digested in nitric acid on a dry block heater at 100°C for one hour, rather than in the digester. No differences were found between digestion methods in the final mineral composition of carcass samples [3]. Finally, digests were diluted with nanopure water for final mineral analysis. All concentrations were determined by mass. Calcium was quantified as a measure of the hydroxyapatite content of bone and sodium was quantified because substantial stores of this element can also be present in bone [28]. The Ca^2+^ and Na^2+^ content of these samples was determined by inductively coupled plasma optical emission spectrometry (Perkin Elmer Optima 7300DV; Waltham, MA, USA) using the following wavelengths: Ca^2+^ -317.93 nm and Na^2+^ - 589.52 nm. All total body composition data was corrected for the composition of the removed femora (mineral content of the femur *2). Total mass, ash, calcium and sodium invested in the litter were based on the average for the 3 individuals analyzed times the litter size at termination of the study.

### Bone Architecture and Strength

The architectural characteristics of the left femora were quantified using micro-computed tomography at the Small Animal Phenotyping Lab at the University of Alabama, Birmingham (MicroCT 40, Scanco Medical, Bassersdorf, Switzerland). All bones were scanned in air. The femoral cortical bone was evaluated based on 25 transverse slices 6.5 mm above the growth plate. The femoral cancellous bone characteristics were based on 100 total transverse slices scanned just proximal to the distal growth plate. All scans were completed in a 12 mm diameter tube with a voxel size of 12 μm^3^. The x-ray tube potential was 55 kVp with an intensity of 145 μA; the integration time was set at 200 ms. Analysis of the architectural properties of the bone were completed with the microCT Evaluation Program supplied by Scanco (v5.0A). All trabecular measurements were completed in three dimensions for the entire volume quantified. Variables presented were based on those recommended by Bouxsein et al [29]. We also included the microCT measurements of bone mineral density for the cortical bone for comparison to prior work but not the measurements of trabecular bone mineral density due to the relative inaccuracy of these values for cancellous bone [29]. Cortical total volume and bone volume were converted to cross-sectional area by dividing each value by # of slices * voxel size, as described by Bouxsein et al [29].

The strengths of the left adult femora were determined using a Mini Bionix Mechanical Testing System (model 858, MTS Systems Corp., Minneapolis, MN). All bones were wrapped in phosphate buffered saline soaked gauze and then frozen at −20°C in air tight tubes after collection for at least 3 days prior to testing break strength [30]. The gauze and overlying muscle tissue were removed immediately prior to testing. Three-point bending tests were completed using monotonic axial displacement with a 100 N load cell. Each bone was centered on two points 10 mm apart. Force was applied to the mid-shaft of the bone at 0.05 mm/s by a third point on the side of the bone opposite the initial points of contact. Maximum force required to break each bone was recorded. The strength of pup bones was analyzed in a similar manner with a different instrument, specifically a TA.XT.PLUS Texture Analyzer (Scarsdale, NY). The three-point bend test was conducted with a 50 N load cell and the bone centered on a 3.1 mm span.

### Mouse Climbing Activity

Climbing activity was monitored each night between 8 PM and 6 AM using a Reconyx game camera (Reconyx, Inc., Holmen, WI). Photos were taken every 5 seconds with the camera positioned to take an image of all towers at once. Photos were merged into a movie using Picasa 3 (Google, Inc, Mountain View, CA) and climbing was quantified with Noldus Ethovision XTsoftware (Noldus Information Technology, Wageningen, Netherlands). Time spent climbing was defined as time visible in the hardware cloth tower. The center point detection setting was used to track the white mouse against the background of the dark room. The mice were detected as a moving white object that was 150–4000 pixels, allowing for changes in size with movement. The videos were sampled at 0.02 frames / s.

### Statistical Analyses

Maternal characteristics, maternal body composition variables, maternal femoral bone variables, litter characteristics, femoral cancellous architecture, femoral cortical architecture, and vertebral cancellous architecture were compared between treatment groups. All variables were tested for normality within treatment group using a Shapiro-Wilk test. Initially, all treatment groups were compared using ANCOVA with litter size at birth included as a covariate. When the covariate was not significant, litter size was dropped from the model and treatment groups were compared with a two-tailed t-test if data were normally distributed, data that were not normally distributed were normalized with a log transformation. In one case, number of pups cannibalized, a log transformation could not be completed because the data included zeros. For this comparison, a two-tailed Wilcoxon test was used to compare groups. All variables compared with ANCOVA were normally distributed. To correct the α-value for multiple comparisons, a sequential Bonferroni correction was used within the categories described above (all listed as subheading in Tables 1 and 2). The average absolute values of all correlations among variables within category were determined and this average was used to correct the Bonferroni adjustment for correlated variables. These adjusted α-values were calculated using the Quantitative Skills webpage (http://www.quantitativeskills.com/). All proportional data were arcsine transformed following a square-root transformation [31]. A single mouse that did not reproduce in the tower group was excluded from all analyses.

**Table 1:** Comparison of maternal characteristics, body composition, femoral bone between treatment groups.

	**Tower**	**Tunnel**	**Stat**	**df**	**P**	**Adj. α**	**Interpret.**
**Maternal characteristics**							
litter size^a^	12.8 ± 0.6	12.7 ± 0.6	t = 0.04	21	0.972	0.050	not sign.
pups cannibalized	0.82 ± 0.40	0.58 ± 0.26	Z = 0.07	---	0.944	0.029	not sign.
body mass (g)	50.2 ± 1.2	50.4 ± 1.6	t = 0.11	21	0.912	0.020	not sign.
food intake (g)	282 ± 14.0	287 ± 7	t = 0.31	14.1^d^	0.760	0.016	not sign.
**Maternal body composition**							
fat (% DM^b^) ^a^	20.5 ± 2.2	28.4 ± 3.3	t = 2.01	21	0.057	0.025	not sign.
ash (% FFDM^c^)	13.1 ± 0.3	13.7 ± 0.3	t = 1.20	21	0.179	0.029	not sign.
Ca (mg/g FFDM) ^a^	30.1 ± 1.0	31.5 ± 1.0	t = 0.93	19	0.366	0.050	not sign.
Na (mg/g FFDM)	3.99 ± 0.15	4.20 ± 0.14	t = 1.05	19	0.306	0.035	not sign.
**Maternal femoral characteristics**							
mass (mg)	51.2 ± 1.4	50.2 ± 1.5	t = 0.47	19	0.644	0.035	not sign.
length (mm) ^a^	17.1 ± 0.2	17.2 ± 0.2	t = 0.83	19	0.415	0.022	not sign.
ash (% FFDM)	61.0 ± 0.6	61.5 ± 0.5	F = 0.45	1	0.509	0.025	not sign.
Ca (% FFDM)	17.6 ± 0.7	17.8 ± 0.7	F = 0.06	1	0.812	0.050	not sign.
Na (% FFDM)	0.875 ± 0.030	0.915 ± 0.034	t = 0.86	19	0.402	0.020	not sign.
strength (N)	18.0 ± 0.9	17.2 ± 1.2	F = 0.34	1	0.565	0.029	not sign.

Means are presented ± se. Results of statistics (Stat) analysed with ANCOVA (F) are given when litter size was a significant covariate (partial F statistic is for treatment group). When litter size was not significant, t-tests (t) were used when the data was normally distributed, log transformed t-tests (t) were used when the data were not normally distributed, or Wilcoxon signed rank test (Z) were used when data were not normally distributed and included zeros (which cannot be log transformed). Sequential Bonferroni correction (adj. α) and interpretation of comparisons are given.

^a^Log_10_ transformed

^b^DM = dry mass

^c^FFDM = fat free dry mass

^d^unequal variance, Satterthwaite method used for comparison

**Table 2:** Comparison of maternal femoral characteristics between treatment groups.

	**Tower**	**Tunnel**	**Stat**	**df**	**P**	**Adj. α**	**Interpret.**
**Femoral cancellous bone**							
bone volume fraction (%)	15.5 ± 1.9	12.3 ± 1.0	F = 3.87	1	0.064	0.031	not sign.
trabecular number (1/mm)	4.07 ± 0.50	2.86 ± 0.23	F = 6.87	12.8^a^	0.017	0.028	**sign.**
trabecular thickness (mm)	0.038 ± 0.001	0.042 ± 0.002	t = 1.76	20	0.084	0.037	not sign.
trabecular spacing (mm)	0.261 ± 0.051	0.337 ± 0.034	t = 1.28	20	0.212	0.050	not sign.
**Femoral cortical bone**							
BMD^b^ (mg/cm^3^)	1367 ± 13.7	1419 ± 4.88	t = 3.56^a^	11.3	0.004	0.016	**sign.**
total area IPE^c^ (mm^2^)	2.80 ± 0.11	2.74 ± 0.09	t = 0.51	20	0.616	0.023	not sign.
bone area IPE (mm^2^)	0.926 ± 0.027	0.920 ± 0.030	t = 0.15	20	0.880	0.050	not sign.
bone area fraction (%)	33.3 ± 1.3	33.8 ± 1.0	F = 0.09	1	0.772	0.031	not sign.
cortical thickness (μm)	56.7 ± 2.3	64.4 ± 2.1	t = 2.45	20	0.024	0.019	not sign.

Means are presented ± se. Results of statistics (Stat) analysed with ANCOVA (F) are given when litter size was a significant covariate (partial F statistic is for treatment group) or t-tests (t) when litter size was not significant. Sequential Bonferroni corrections (adj. α) and interpretation of comparisons are given.

^a^unequal variance, Satterthwaite method used for comparison

^b^bone mineral density

^c^inside periosteal envelope

## Results

Mice in the tower treatment group were successfully encouraged to climb with lactating mice spending 15.0±3.2 (s.e.m) min per night in the tower. The tunnel treatment was also successful. Although movements could not be quantified, mice were regularly seen moving back and forth within the tunnels. Mice in the tunnel group were rarely seen hanging from the box lids.

There were no differences between groups in litter size at birth or number of pups cannibalized. There was also no difference between groups in cumulative food intake by lactating mothers or maternal body mass, body fat, total body ash content, total body calcium or sodium content of mothers at peak lactation (Table 1). Femoral mass, dimensions, ash content and calcium and sodium content were also similar between groups (Table 1). In addition, there was no difference between groups in relative maternal investment based on litter mass and litter skeletal size (Table 1). There was no difference between groups in the breaking strength of the femora (Table 1).

**Fig 2:**
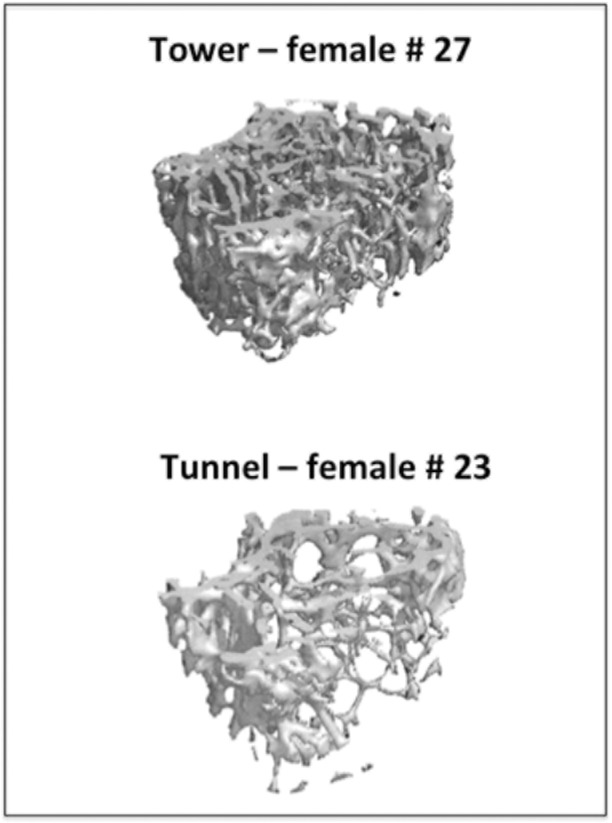
MicroCT image of femoral trabecular bone for a tower and tunnel mouse. Notice the difference in trabecular density between the mice. Each mouse reared 15 offspring.

There were differences between treatment groups in the architecture of the cancellous and cortical bone within the femora of the mice. For cancellous bone, number of femoral trabeculae was greater in the tower than the tunnel (Table 2, Fig. 2). For the cortical bone, mineral density midshaft was significantly lower in the tower group than the tunnel group and there was a trend suggesting that cortical thickness was also lower but this was not significant when adjusted for multiple comparisons (Table 2).

Finally, there was no difference between groups in the amount of energy (based on body mass) or the total amount of mineral invested in reproduction (Table 3). However, there was a significant difference in pup body composition between groups. Specifically, pups born to mothers in the tower group had significantly higher concentration of calcium their body than pups born to mothers in the tunnel groups (Table 3). The body mass, body fat, and total sodium content of the young was not different but total body ash displayed a trend that is consistent with pups in the tower group having more mineral. This difference in pup quality was not reflected in their femurs that displayed no difference in mass, composition or strength between groups (Table 3).

**Table 3:** Comparisons maternal investment, pup body composition and pup femoral characteristics between treatment groups.

	**Tower**	**Tunnel**	**Stat**	**df**	**P**	**Adj. α**	**Interpret.**
**Estimated investment by mother in litter**							
total mass (g)	104 ± 5	104 ± 5	t = 0.06	20	0.954	0.050	not sign.
total ash (g)	3.77 ± 0.27	3.29 ± 0.23	t = 1.39	20	0.180	0.035	not sign.
total Ca (g)^a^	0.721 ± 0.038	0.659 ± 0.045	t = 1.05	20	0.306	0.038	not sign.
total Na (g) ^a^	0.121 ± 0.010	0.119 ± 0.008	t = 0.20	20	0.846	0.042	not sign.
**Pup body composition**							
body mass (g) ^a^	8.96 ± 0.44	8.67 ± 0.62	F = 0.16	1	0.700	0.05	not sign.
fat (% DM)^b^	24.8 ± 2.9	21.2 ± 2.3	F = 0.85	1	0.367	0.022	not sign.
ash (% FFDM^c^) ^a^ ^,^ ^d^	15.2 ± 0.9	13.2 ± 0.4	t = 2.14	14.6	0.050	0.018	not sign.
Ca (% FFDM)	2.87 ± 0.06	2.65 ± 0.06	F = 6.64	1	0.012	0.015	**sign.**
Na (% FFDM) ^a^	0.480 ± 0.021	0.493 ± 0.022	t = 0.47	20	0.646	0.030	not sign.
**Pup femoral characteristics**							
bone mass (mg)	8.62 ± 0.62	7.96 ± 0.69	F = 0.38	1	0.547	0.022	not sign.
length (mm)	8.99 ± 0.13	8.99 ± 0.20	F = 0.08	1	0.782	0.050	not sign.
ash (% FFDM)	50.3 ± 0.9	48.6 ± 1.74	F = 0.57	1	0.459	0.015	not sign.
Ca (% FFDM	17.2 ± 0.3	16.9 ± 0.5	t = 0.62	20	0.541	0.018	not sign.
Na (% FFDM) ^a^	1.52 ± 0.4	2.27 ± 1.00	t = 0.49	20	0.632	0.030	not sign.
strength (N) ^a^	3.28 ± 0.26	2.96 ± 0.34	F = 0.93	1	0.347	0.013	not sign.

Means are presented ± se. Results of statistics (Stat) analysed with ANCOVA (F) are given when litter size was a significant covariate (partial F statistic for treatment group). When litter size was not significant, t-tests (t) were used when the data was normally distributed, log transformed t-tests (t) were used when the data were not normally distributed, or Wilcoxon signed rank test (Z) were used when data were not normally distributed and included zeros (which cannot be log transformed). Sequential Bonferroni correction (adj. α) and interpretation of comparisons are given.

^a^Log_10_ transformed

^b^DM = dry mass

^c^FFDM = fat free dry mass of body or femur

^d^unequal variance, Satterthwaite method used for comparison

## Discussion

In response to increased strain on the skeleton, we predicted that female mice that climbed regularly during reproduction would retain more bone mineral than females whose movements are limited to a horizontal plane. Results of experimental manipulation of the locomotor environment of mice failed to support this prediction. There was no difference between mineral content of the femora or whole bodies of mothers that primarily travelled in the vertical or the horizontal plane. These observations suggest that the mobilization of bone that occurs during reproduction may be prioritized over the anabolic action stimulated by an increase in loading on the skeleton. Instead of building more bone, reproductive mice subjected to climbing altered the architecture of their bone and transferred more mineral to their offspring.

There was no difference in bone, body ash, or mineral content for lactating mice between treatment groups. Thus, the greater strain associated with climbing relative to walking or running did not counter the loss of mineral from long bones, which commonly occurs during reproduction [6,7,32,33]. Instead, changes in bone architecture suggest that bone was redistributed in response to the strain experienced during locomotion. Specifically, at peak lactation the femora of climbing females had a greater number of trabeculae than the tunnel females, whereas the tunnel females had greater bone density mid-shaft than climbing females. Fracture tests on these bones indicated that the change in architecture did not impact bone strength. Under the condition of this experiment, the mice compensated for loading on bone in a manner that did not reduce bone mineral allocation to their young.

Somatic tissue is used as a nutritional resource under numerous conditions, including reproduction [4,34,35], migration [36,37], hibernation [38,39], and undernourishment [40]. The cost of using somatic tissue during reproduction may come prior to reproduction associated with the accretion of new tissue (or prior to lactation in cases were tissue is depositing during gestation), following reproduction during tissue reconstruction, during mobilization and/or prior to recovery of lost tissues associated with the cost of catabolism and side-effects of reduced body condition. We currently understand little with regard to the tissue-specific constraints placed on mobilization. These constraints undoubtedly vary by species, life history stages, an animal’s physiological condition, its environment and exogenous resource availability, just as reproductive effort is impacted by similar variables.

In the present study, reproductive females that climbed were expected to prioritize self-maintenance, and thus the opportunity for future reproduction, over the current reproductive effort [41,42]. Specifically, mothers that had to climb were predicted to retain a greater proportion of mineral in their load-bearing bones than individuals that moved in the horizontal plane. This, in turn, would have reduced the amount of mineral that could be mobilized to the litter. Yet, the results of this investigation suggest that current reproductive effort was prioritized over self-maintenance and potential for future reproductive performance. Although females in the tower group reared pups that had a significantly higher concentration of total body calcium than females in the tunnel group, data on the relative calcium composition of mothers, maternal food intake, and the total amount of calcium in the litter (by mass) did not reflect this difference. Thus, we suspect that this effect may not reflect greater calcium intake by the pups born to climbing mothers. Future studies should evaluate whether the level or form of activity displayed by mothers impacts the efficiency of bone mineralization in the young. Hormones or other bioactive factors transferred to young in utero or via milk could be responsible for this effect [43].

The residual reproductive value (RRV) hypothesis states that in iteroparous species, females should adjust their current reproductive expenditure relative to the number of offspring that they are likely to produce in the future [44,45]. In general, reproductive effort is expected to increase with age as a female’s RRV declines ([45,46] but see [47]). Yet, young breeders can have high self-maintenance requirements if they are still growing and they can be less efficient at partitioning resources than older females. As a result, the probability of survival can be lower in younger than middle-aged females [48]. When reduced probably of survival is taken into account, the RRV of young mothers can be low, just as it is in old females [44,48,49]. All mice in the present study were primiparous. It is possible that due to their age and inexperience, the results reflect a reproductive strategy that assumes a low probability of future reproduction for young, primiparous individuals. Changes in bone mobilization relative to age and form of locomotion are worthy of further consideration.

Alternative explanations for the results of this study include the conditions of the experiment were insufficient to simulate bone anabolism or bone anabolism was enhanced in the tunnel group, and the sample size and values quantified were insufficient to detect differences between groups. It must be assumed that mice in the tower group experienced sufficient strain to stimulate skeletal anabolism because the set-up was similar and the duration of the experiment more than twice as long (4 verses 10 weeks) as prior work that found that climbing had anabolic effects on cortical and trabecular bone in non-reproductive mice. Although the tower height in the present study was 40 cm lower (60 cm verses 100 cm) than the tower made available to non-reproductive mice in the study by Mori et al. [12], the mass of the mice and time spent climbing, and not the height of the wall the animal climbed, is relevant to the amount of mechanical loading that it experienced. Climbing mice in the present study both spent more time climbing (15 min / 10 hour period verses 12 min / 24 hr. period in the non-reproductive mice) and were 2–3 times heavier than the non-reproductive mice (up to 75 g prior to parturition and 50 g at termination of the experiment verses 25 g in the non-reproductive mice) describe by Mori et al [12]. In the study by Mori et al. [12], control animals were maintained in a standard size mouse box, much like the third group in this investigation that was excluded from analysis. No attempt was made to model or measure differences in the mechanical loading experienced by animals in the tower and tunnel group. Thus, the possibility that strain did not differ between groups cannot be ruled out.

In this study, twelve mice were assigned to each treatment group. One mouse from the tower group failed to breed, and one bone sample from each of the groups was lost due to error during analysis. Thus samples sizes range from 10–11 per treatment group. In Mori et al.’s [12] investigation, approximately 13 mice were assigned to each treatment group and in Peng’s [32] work on the effects of litter size on bone mobilization in rats, difference in maternal bone mineral content were established with only 4–7 animals per treatment group. The sample sizes selected for this study were typical for this type of investigation. Nevertheless, it remains possible differences between groups that could not be detected due to insufficient statistical power. Despite the fact that the litter size, cumulative body mass, and cumulative body lengths of the litters did not differ between groups, it also remains possible that there were undetected differences in the amount of mineral transferred to young.

Because recent work has shown that energy and bone metabolism interact, the control group (i.e. the tunnel group) was designed to have enhanced energy expenditure over a standard mouse box in effort to hold energy expenditure constant. Because food intake was comparable between groups, it is clear that this effort was successful. Bone metabolism has been shown to interact with energy metabolism through the positive impacts of osteocalcin (produced during bone formation) on insulin production [26] and negative impact of leptin on bone formation and the positive impact of leptin on relative bone resorption [24]. Leptin production is normally increased with increased food intake, but this is uncoupled during lactation, when leptin levels remain low [50,51]. Our understanding of the interactions between bone and energy metabolism are still in their infancy. How these processes scale up to the whole individual, particularly during different life history events, is not well understood. It is possible similarities in energy expenditure stimulated relatively comparable patterns of cumulative turnover between groups.

## Conclusions

Under the conditions of this experiment, strain on the skeleton during locomotion did not impact bone mineral allocation by mothers to their young. Instead, strain on the skeleton during reproduction appeared to stimulate a redistribution of bone in response to the stresses it experienced. Although our results suggest that the pups of climbing females display greater relative calcium content, we do not believe that this reflects difference in mineral allocation between groups. Free-ranging animals are likely to experience greater loading during natural movements between their nests and food resources than was experienced by any of the mice in this study. Thus, the results of this study also do not rule out the possibility that the anabolic effects of loading and catabolic effects of mineral mobilization interact in reproductive females.
